# Identification of proteases employed by dendritic cells in the processing of protein purified derivative (PPD)

**DOI:** 10.1186/1476-8518-2-8

**Published:** 2004-08-02

**Authors:** Mansour Mohamadzadeh, Hamid Mohamadzadeh, Melissa Brammer, Karol Sestak, Ronald B Luftig

**Affiliations:** 1 Department of Microbiology, Immunology and Parasitology, Louisiana State University Health Sciences Center, New Orleans, LA, USA; 2 Johannes Wolfgang Goethe Medical School, Frankfurt, Germany; 3 Tulane National Primate Research Center Science, New Orleans, Louisiana, USA; 4 Tulane Medical School, New Orleans, LA, USA

## Abstract

Dendritic cells (DC) are known to present exogenous protein Ag effectively to T cells. In this study we sought to identify the proteases that DC employ during antigen processing. The murine epidermal-derived DC line Xs52, when pulsed with PPD, optimally activated the PPD-reactive Th1 clone LNC.2F1 as well as the Th2 clone LNC.4k1, and this activation was completely blocked by chloroquine pretreatment. These results validate the capacity of XS52 DC to digest PPD into immunogenic peptides inducing antigen specific T cell immune responses. XS52 DC, as well as splenic DC and DCs derived from bone marrow degraded standard substrates for cathepsins B, C, D/E, H, J, and L, tryptase, and chymases, indicating that DC express a variety of protease activities. Treatment of XS52 DC with pepstatin A, an inhibitor of aspartic acid proteases, completely abrogated their capacity to present native PPD, but not trypsin-digested PPD fragments to Th1 and Th2 cell clones. Pepstatin A also inhibited cathepsin D/E activity selectively among the XS52 DC-associated protease activities. On the other hand, inhibitors of serine proteases (dichloroisocoumarin, DCI) or of cystein proteases (E-64) did not impair XS52 DC presentation of PPD, nor did they inhibit cathepsin D/E activity. Finally, all tested DC populations (XS52 DC, splenic DC, and bone marrow-derived DC) constitutively expressed cathepsin D mRNA. These results suggest that DC primarily employ cathepsin D (and perhaps E) to digest PPD into antigenic peptides.

## Review

Dendritic cells (DC) are professional antigen presenting cells that induce primary antigen specific T cell responses [1] and exhibit all functional properties required to present exogenous antigen (Ag) to immunologically naïve T cells. These properties include: a) uptake of exogenous Ag via receptor-mediated endocytoses, b) processing of complex proteins into antigenic peptides, c) assembly of these peptides with MHC molecules, d) surface expression of MHC molecules as well as costimulatory molecules, including CD80, CD86, and CD40, e) secretion of T cell stimulatory cytokines, including IL-1β, IL-6, IL-8, TNF-α, and macrophage inflammatory protein (MIP)-1α and f) migration into draining lymph nodes [2].

In the present study, we sought to characterize the Ag processing capacity of DC, as well as the enzymes previously involved in this process. In this regard, several groups have previously reported that epidermal LC and splenic DC, both of which contain small numbers of non-DC contaminants, exhibit significant Ag processing capacities [3-12]. LC freshly obtained from skin are quite potent in their Ag processing capacity, but the majority of these LC lose this capacity as they "mature" during subsequent culture [3-6,12]. On the other hand, other reports have shown that DC are less efficient than macrophages in Ag processing, with each employing different pathways for Ag processing [10,13-16]. These differences suggest the possibility of unique pathways and requirements for Ag presentation by DC.

With respect to the mechanisms by which DC process complex protein Ags, chloroquine has been shown to inhibit this process; this suggests that Ag processing primarily occurs within acidic compartments [6-8], [10-12]. Macrophages and B cells have been reported to employ cathepsins B, D, and/or E for digesting protein Ag, including ovalbumin (OVA), hen egg white lysozyme (HEL), myoglobin, exogenous IgG, and *Staphylococcus aureus *nuclease [17-35]. These proteases may each exhibit differential pathways for activity; for example, macrophages appear to employ cathepsin D for the initial cleavage of myoglobin and cathepsin B for C-terminal trimming of resulting fragments [17]. Little information, however, has been available with respect to proteases that are employed by DC for Ag processing. Thus, in the present study we sought to define the protease profiles produced by DC and then to identify which protease(s) would primarily mediate Ag processing in DC.

## Materials and Methods

### Cells

The XS52 DC cell line (a gift of Dr. Takashima, Dallas, Texas), a long-term DC line established from the epidermis of newborn BALB/c mice [23], were expanded in complete RPMI in the presence of 1 ng/ml murine rGM-CSF and 10% culture supernatants collected from the NS stromal cell line as described previously [23]. Other phenotypic and functional features of this line are descibed elsewhere [23-25]. As responding T cells, we used the protein purified derivative (PPD)-reactive Th1 clone LNC.2F1 and the Th2 clone LNC.4K1 [26], both of which were kindly provided by Dr. E. Schmitt (Institute for Immunology, Mainz, Germany). As control cells, we also employed Pam 212 keratinocytes [27], 7–17 dendritic epidermal T cells (DETC) [28], J774 macrophages (ATCC, Rockville, MD), and BW5147 thymoma cells (ATCC).

Splenic DC were purified from BALB/c mice (Jackson Laboratories, Bar Harbor, ME) by a series of magnetic bead separations as before [24,25]. Briefly, spleen cell suspensions were first depleted of B cells using Dynabeads conjugated with anti-mouse IgG. Subsequently, T cells were removed using beads coated with anti-CD4 (GK1.5) and anti-CD8 mAbs (3.155), and then macrophages were depleted using beads conjugated with F4/80 mAb. Finally, DC were positively sorted using beads coated with anti-DC mAb 4F7 [29]. The resulting preparations routinely contained > 95% DC, as assessed by flow cytometry. DCs were propagated from bone marrow as described by Inaba et al. [30]. Using magnetic beads, bone marrow cell suspensions were first depleted of B cells (with anti-mouse IgG), I-A^+ ^cells (with 2G9 mAb, Pharmingen, San Diego, CA), and T cells (with GK1.5 and 3.155 mAbs). The remaining I-A^- ^cells were then cultured in the presence of GM-CSF (10 ng/ml). The purity of bone marrow derived DC was more than 95% as determined by flow cytometry using anti-CD11c and anti-I-A antibody (not shown).

### Determination of protease activities

Cells were lysed in 0.1% Triton X-100 in 0.9% NaCl; extracts were then examined for protease activities using the following substrates: a) Z-Arg-Arg-βNA (for cathepsin B, at pH 6.0), b) denatured hemoglobin (cathepsin D/E, pH 3.0), c) Arg-βNA (cathepsin H, pH 6.8), d) Z-Phe-Arg-MCA (cathepsin J, pH 7.5), e) Z-Phe-Arg-MCA (cathepsin L, pH 5.5), f) Gly-Phe-βNA (DPPI or cathepsin C, pH 5.5), g) BLT ester (BLT esterase, pH 7.5), and h) Suc-Ala-Ala-Pro-Phe-SBz and Suc-Phe-Leu-Phe-SBz (chymotrypsin-like proteases, pH 7.5). Samples were incubated at the indicated pH and enzymatic activities were assessed by colorimetric or fluorogenic changes [31]. Enzymatic activities were expressed as nmol/min/mg soluble protein, in which protein concentrations were measured by the bicinchoninic acid method using bovine serum albumin as a standard [32].

### Ag presentation and T cell stimulation assays

XS52 DC were γ-irradiated (2000 rad) and then pulsed for 8 hr with 100 μg/ml of PPD (kindly provided by Dr. E. Schmitt, Mainz, Germany) in the presence of each of the following inhibitors (or vehicle controls): a) pepstatin A (100 μg/ml, Sigma, St. Louis, MO), b) DCI 100 μM, Sigma), c) E-64 (100 μM, Sigma), d) DMSO (1%), and e) NH_4_CL (15 mM). Subsequently, the XS52 cells were washed 3 times with PBS to remove unbound PPD and then cultured in 96 round-bottom well-plates (10^4 ^cells/well) with either the PPD-reactive Th1 or Th2 clone (10^5 ^cells/well) in the presence of the same inhibitor at the above concentration. In some experiments, XS52 DC were pulsed overnight with PPD in the presence of an inhibitor and then fixed with 0.05% glutaraldehyde in PBS for 30 seconds at 4°C; the fixation reaction was stopped by adding 0.1 M L-lysine. These XS52 cells were then washed with PBS and examined for their ability to activate Th1 or Th2 clones in the absence of protease inhibitors. In order to determine the mechanism of action for pepstatin A, XS52 cells were pulsed in its presence with PPD either in a native form or following digestion with trypsin-conjugated sepharose beads (Pierce, Rockford, IL) for 15 minutes at 37°C. We also examined the effect of added pepstatin A on the capacity of XS52 cells to activated allogeneic T cells isolated from CBA mice (Jackson Laboratories). Samples were pulsed for 18 hr with 1 μCi of ^3^H-thymidine and then harvested using an automated cell harvestor.

### RT-PCR Analysis

mRNA expression for cathepsin D was examined by RT-PCR. RNA isolation, reverse-transcription, and cDNA amplification were carried out as previously described [33]. The following primers were designed based on the published sequence of murine cathepsin D [34]: 5'-GGTCAGAGCAGGTTTCTGGG-3' and 5'-GCTTTAAGCTTTGCTCTCTTCGGG-3'. After 25 cycles of amplification, PCR products were analyzed in 1% agarose gel electrophoresis containing 2 μg/ml ethidium bromide. Other experimental conditions, including primer sequences for the β-actin control, are described elsewhere [33].

## Results

### DC exhibit several different protease activities and they process the complex protein Ag PPD into antigenic peptides

In the first set of experiments we sought to identify which protease activities were expressed by DC. A panel of synthetic peptide and protein substrates was incubated with extracts prepared from three DC populations: the XS52 DC line, 4F7^+ ^splenic DC, and GM-CSF-propagated bone marrow DC. As noted in Table 1, each DC population exhibited all tested protease activities, including cathepsins B, C, D/E, H, J, and L, BLT esterase, and chymotrypsin-like proteases. Each protease activity in DC was substantially higher (up to 20 fold) than that detected in the BW5147 thymoma cell line, a line that expresses relatively low levels of protease activities. Moreover, cathepsin D/E activity was undetectable (<1 nmol/min/mg) in Pam 212 keratinocytes and 7–17 DETC (data not shown), indicating further cell type-specificity. These results demonstrate that DC produce a variety of protease activities and at relatively high levels.

**Table 1:** Protease Profiles Expressed by Several DC Populations

**Protease**	**XS52 DC**	**Splenic DC**	**Bone Marrow DC**	**BW5147 Thymoma Cells**
Cathepsin B^1^	133 ± 39^2^	123 ± 3.3	121 ± 3.3	1.5 ± 0.08
Cathepsin C	34 ± 7	16 ± 4	0.4 ± 0.1	<0.01
Cathepsin D/E	34 ± 6	30 ± 14	22 ± 2	1.6 ± 0.1
Cathepsin H	2.8 ± 0.8	3.5 ± 0.7	1 ± 0.2	0.9 ± 0.2
Cathepsin J	26 ± 0.3	0.7 ± 0.3	3.0 ± 0.1	0.2 ± 0.09
Cathepsin L	14 ± 0.6	26 ± 11	19 ± 0.6	0.6 ± 0.08
BLT esterase	25,000 ± 400	58,000 ± 4,000	21,300 ± 100	<100
Suc-FLF-SBz esterase	1,900,000 ± 61,000	310,000 ± 10,800	1,150,000 ± 10,200	12,000 ± 100
Suc-AAPFSBZ esterase	433,000 ± 2,900	134,000 ± 7,300	360,000 ± 6,100	3,400 ± 600

^1^Extracts prepared from the indicated cell types were examined for protease activities using a panel of standard substrates. ^2^Enzymatic activities are expressed as nmol/min/mg soluble protein. Data shown represent the mean ± SD from three independent preparations.

**Figure 1:**
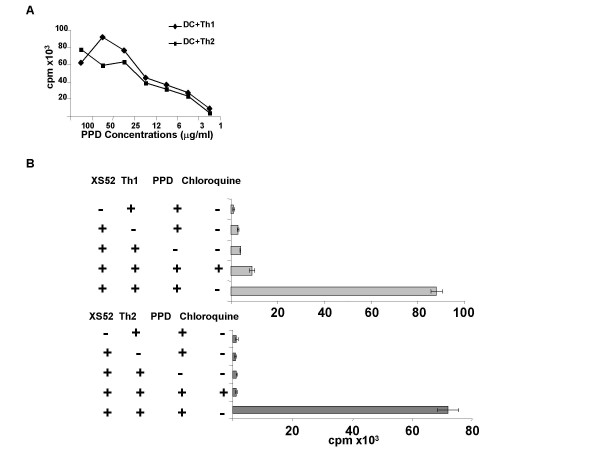
**XS52 DC are capable of presenting native PPD effectively to T cells: **(A) XS52 DC were γ-irradiated and then pulsed for 8 hr with the indicated concentrations of PPD. The PPD-reactive Th1 clone (diamonds) or Th2 clone (squares) (10^5 ^cells/well) was cultured for 2 days with PPD-pulsed XS52 cells (10^4 ^cells/well). (B) Following a 3 hr incubation with or without chloroquine (100 μM), XS52 DC were pulsed with PPD (100 μg/ml) in the presence or absence of chloroquine (100 μM) and then examined for their capacity to activate the PPD-specific Th1 and Th2 clones. Data shown are the mean ± SD (n = 3) of ^3^H-thymidine uptake. Baseline proliferation of γ-irradiated XS52 DC alone was <300 cpm.

We next asked whether DC are capable of digesting a complex protein Ag into antigenic products. In this regard, it has been reported previously that the original XS52 DC, as well as clones derived from this line, are capable of presenting KLH to the KLH-specific Th1 clone HDK-1 [23]. These results, however, did not fully test the processing capacity because it remained uncertain whether the conventional KLH preparation, which also contained many small molecular weight species, was indeed "processed" before effective presentation. For this reason, we developed a new experimental system using two PPD reactive T cell clones, a Th1 clone LNC.2F1 and a Th2 clone LNC.4K1. The advantage of PPD lies in the relative certainty of its purity. When pulsed with native PPD for 8 hr, XS52 DC were capable of stimulating both T Cell clones effectively. In dose-response experiments (Fig 1A), XS52 DC induced maximal activation of both T cell clones at 25–100 μg/ml of PPD, whereas no significant activation was observed, even at higher concentrations, in the absence of XS52 DC (data not shown). Importantly, chloroquine (100 μM) inhibited completely the capacity of XS52 cells to activate both Th1 and Th2, T cell clones (Fig. 1B), indicating the requirement for processing of PPD in an acidic environment. These observations indicate that XS52 DC do possess the capacity to digest a complex protein Ag into an immunogenic Ag.

### Pepstatin A inhibits the capacity of XS52 DC to present native PPD to T cells

**Figure 2:**
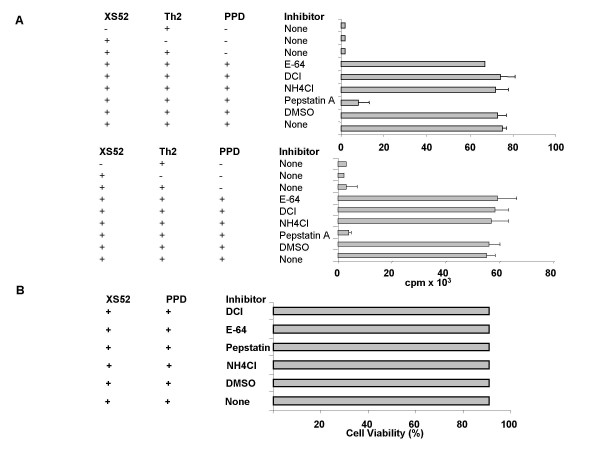
**Pepstatin A inhibits the capacity of XS52 DC to present native PPD: **(A) γ-irradiated XS52 DC were pulsed with PPD (100 μg/ml) in the presence or absence of each protease inhibitor (100 μg/ml pepstatin A, 100 μM DCI, or 100 μM E-64) or vehicle alone (1% DMSO or 15 mM NH_4_Cl). XS52 DC were then cultured for 2 days with the PPD-reactive Th1 or Th2 clone in the continuous presence of the same inhibitor or vehicle alone. Data shown are the mean ± SD (n = 3) of ^3^H-thymidine uptake in three representative experiments. (B) XS52 DC were incubated with each of protease inhibitor (100 μg/ml pepstatin A, 100 μM DCI, or 100 μM E-64) or vehicle alone (1% DMSO or 15 mM NH_4_Cl) for 16 hrs. Subsequently, cells were harvested and their viability was measured by trypan blue.

**Figure 3:**
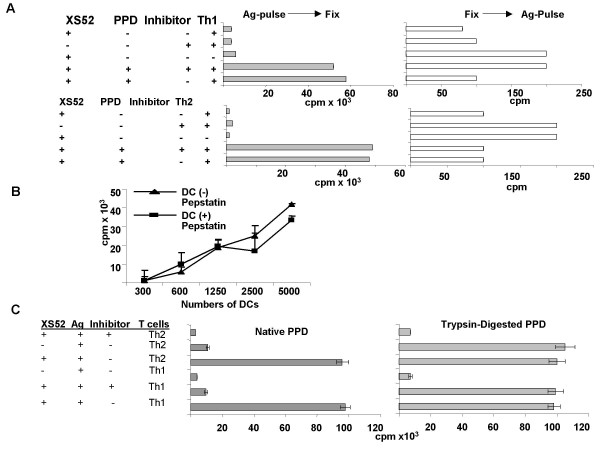
**Failure of pepstatin A to inhibit the Ag presenting capacity of PPD-pulsed and fixed XS52 DC: **(A) γ-irradiated XS52 DC were pulsed with PPD and then fixed with paraformaldehyde (left panels). Alternatively, XS52 DC were first fixed and then pulsed with PPD. Subsequently, the XS52 DC were cultured with the PPD-specific Th1 or Th2 clone in the presence or absence of pepstatin A. Data shown are the mean ± SD (n = 3) of ^3^H-thymidine uptake. (B): Allogeneic splenic T cells isolated from CBA mice (5 × 10^5 ^cells/well) were cultured for 4 days with the indicated numbers of γ-irradiated XS52 DC in the presence or absence of pepstatin A. Data shown are the mean ± SD (n = 3) of ^3^H-thymidine uptake. (C): γ-irradiated XS52 DC were pulsed for 8 hr with either native PPD or trypsin-digested PPD in the presence or absence of pepstatin A. XS52 DC were then cocultured for 4 days with PPD-reactive Th1 or Th2 clones in the presence or absence of pepstatin A. Cocultures were then pulsed for 18 hr with ^3^H-thymidine and then harvested using a β-counter.

To identify the proteases responsible for processing PPD, we employed three inhibitors: pepstatin A (aspartic acid protease inhibitor), DCI (serine protease inhibitor), and E-64 (cysteine protease inhibitor). XS52 DC were pulsed for 8 hr with native PPD in the presence of each inhibitor and then examined for the capacity to activate PPD-reactive Th1 and Th2 clones. To ensure a maximal effect, inhibitors were also added to cocultures of XS52 DC and T cells. As noted in Figure 2A, pepstatin A (100 μg/ml) almost completely blocked the capacity of XS52 cells to stimulate both T cell clones. When XS52 DC were pretreated with pepstatin A only during the 8 hr of Ag pulsing (but not during the subsequent coculture with T cells), we also observed significant, albeit less effective, inhibition (data not shown). By contrast, neither DCI nor E-64 caused any significant inhibition (Figure 2A). No inhibition was observed after treatment with 1% DMSO or 15 mM ammonium chloride alone, which was used to dissolve the above inhibitors. With respect to the mechanism of pepstatin A inhibition, the XS52 DC remained fully viable after 8 hr pre-incubation with pepstatin A (Figure 2B), thus excluding the possibility that pepstatin A had simply killed the XS52 DC. When pepstatin A was added to XS52 DC that had been pulsed with PPD and then fixed with paraformaldehyde, no inhibition was observed (Figure 3A). Moreover, pepstatin A failed to affect the capacity of XS52 DC to stimulate allogeneic T cells in a primary mixed lymphocyte reaction (Figure 3B); making it unlikely that pepstatin A had impaired the T cell-stimulatory capacity of XS52 DC. Finally, pepstatin A treatment was only effective when the native form of PPD was used as complex Ag, whereas it caused no inhibition when trysin-digested PPD fragments were employed (Figure 3C). Based on these observations, we concluded that pepstatin A had primarily inhibited the processing events.

### Functional role of cathepsin D/E in the processing of PPD by XS52 DC

**Figure 4:**
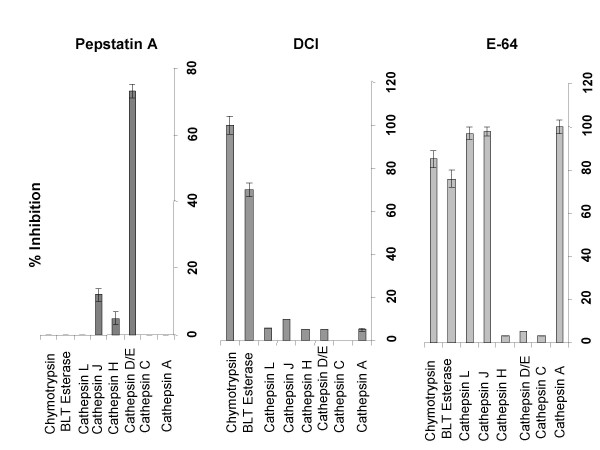
**Pepstatin A Inhibits selectively the cathepsins D/E. **XS52 DC were pretreated for 60 min with each of protease inhibitors or vehicles. After extensive washing, the cells were extracted and subsequently examined for protease activities. Data shown are % inhibition compared with untreated control cells.

To identify the protease(s) inhibited by pepstatin A, XS52 DC were pretreated for 1 hr with pepstatin A (100 μg/ml), and extracts prepared from these cells were then examined for enzymatic activities. As noted in Figure 4, 1 hr pretreatment with pepstatin A was sufficient to block cathepsin D/E activity significantly (>70%). Pepstatin A also inhibited, albeit less effectively, cathepsin J activity and it had no significant effect on other tested protease activities. On the other hand, DCI and E64 were highly inhibitory of the chymotrypsin-like activities as well as cathepsin B, J, and/or L activities, but they did not inhibit cathepsin D/E. These results corroborate previous reports that pepstatin A inhibits cathepsin D/E activity relatively selectively [35]. Thus, it appears that cathepsin D/E is the primary target of pepstatin A, with the implication that these proteases play important roles in processing PPD by XS52 DC.

**Figure 5:**
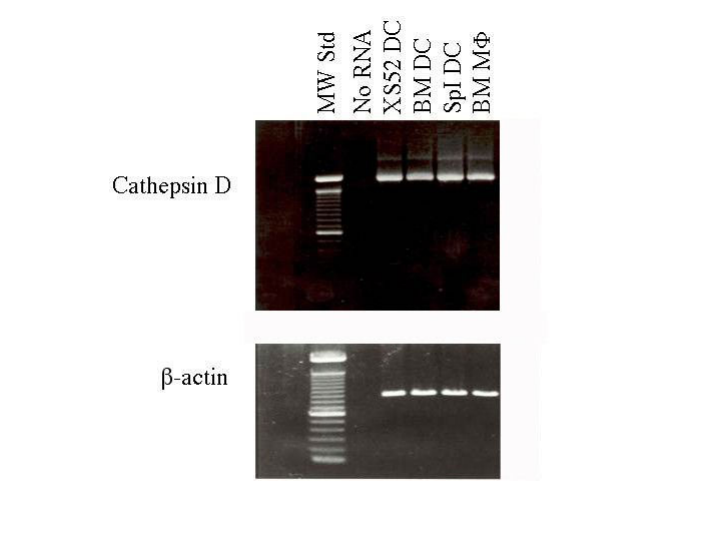
**DC constitutively express cathepsin D mRNA. **Total RNA isolated from the indicated cell types were subjected to RT-PCR analysis for cathepsin D and β-actin. Data are shown, including bone marrow DC and macrophages, as well as 4F7^+ ^splenic DC (splDC), products after 25 cycles of amplification.

Cathepsins D and E are prototypic aspartic acid proteases, which exhibit maximal enzymatic activities at acidic pH. Because both digest denatured hemoglobin effectively, the substrate used to measure cathepsin D/E activity, and because both are equally susceptible to pepstatin A treatment, it remained uncertain where processing of PPD in XS52 DC was mediated by cathepsin D, or cathepsin E, or both. As a first step to answer this question, we detected cathepsin D mRNA by RT-PCR in the XS52 DC line, as well as in 4F7^+ ^splenic DC and a bone marrow derived DC line, indicating that DC do possess the capacity to produce cathepsin D (Figure 5).

## Conclusion

The experiments reported in this study provide new information with respect to complex Ag processing by DC. First, the long-term DC line, XS52 DC, was capable of processing PPD into immunogenic peptides, in the complete absence of other cell types. Although previous studies using several different DC preparations have documented similar results (3–12), this is the first report validating the Ag processing capacity of DC, in the absence of contaminating cells. Second, we have characterized the protease profiles expressed by DC. XS52 DC, 4F7^+ ^splenic DC, and bone marrow-derived DC, all exhibited significant protease activities for cathepsins B, C, D/E, H, J, and L, BLT esterase, and chymotrypsin. Thus, DC possess the capacity to produce a family of protease activities. Finally, pepstatin A, but not other protease inhibitors, abrogated almost completely the ability of XS52 DC to digest native PPD into an antigenic product, suggesting an important role for pepstatin A-sensitive proteases (most likely cathepsin D and/or E) during Ag processing by DC. Taken together, these results reinforce the concept that DC are fully capable of processing complex protein Ag into antigenic peptides.

As described before, macrophages and B cells have been reported to employ cathepsins B, D, and E primarily to digest complex protein Ag, such as ovalbumin (OVA), hen egg white lysozyme (HEL), myoglobin, exogenous IgG, and *Staphylococcus aureus *nuclease (17–22). Here we report that DC also employ cathepsin D and/or E to digest PPD into an immunogenic Ag-product. This conclusion is supported by several lines of evidence: a) pepstatin A, but not other protease inhibitors, completely blocked the presentation of intact PPD by XS52 DC to PPD-reactive Th1 and Th2 clones, whereas it did not affect the presentation of PPD fragments; b) pepstatin A pretreatment inhibited cathepsin D/E activity selectively among the DC-associated protease activities; and c) all tested DC preparations expressed cathepsin D mRNA constitutively. In this regard, DC isolated from the mouse thoracic duct have been reported to produce neglible, if any, cathespin D immunoreactivity (assessed by immunofluorescence staining), whereas peritoneal macrophages produced relatively large amounts [14]. Also comparable levels of cathepsin D/E activity were detected in extracts from bone marrow-derived DC and from bone marrow-derived macrophages (data not shown). This discordance may reflect differences in the DC preparations tested and/or in the assays employed to detect cathepsin D. Nevertheless, our observations indicate that DC employ cathepsin D/E to degrade some protein Ag, with the implication that pepstatin A and other cathepsin D/E inhibitors [36] may be useful to prevent and even to treat unwanted hypersensitivity reactions against such protein Ag.

It is important to emphasize that different protein Ag may be degraded by different proteases in DC. Moreover, DC isolated from different tissues or in different maturational states may employ different proteases. For example, murine DC isolated from the thoracic are unable to digest human serum albumin effectively [14], and murine splenic DC purified following overnight culture have failed to degrade KLH significantly into a TCA-soluble form [13]. Moreover, several reports document that LC lose their Ag processing capacity as they mature in culture [3-6,12]. Thus, it will be interesting to compare DC from different tissues and in different states of maturation for their protease profiles and susceptibilities to pepstatin A treatment. We believe that the experimental system described in this report will provide unique opportunities to study the function of proteases and the regulation of their production in DC.

## Competing Interests

None declared.

## Author's Contributions

Dr. Mohamadzadeh is the major contributor (15%) of the experimental data and a rough draft of the paper. The next three intermediate authors' contributed remaining data and advice. Dr. Luftig was the overall individual who directed the several drafts and contributed to providing a new set of references to the manuscript.
